# Prevalence and distribution of *Gardnerella vaginalis* subgroups in women with and without bacterial vaginosis

**DOI:** 10.1186/s12879-017-2501-y

**Published:** 2017-06-05

**Authors:** Migle Janulaitiene, Virginija Paliulyte, Svitrigaile Grinceviciene, Jolita Zakareviciene, Alma Vladisauskiene, Agne Marcinkute, Milda Pleckaityte

**Affiliations:** 1 Institute of Biotechnology, Vilnius University, Sauletekio al. 7, 10257 Vilnius, Lithuania; 2 National Public Health Surveillance Laboratory, Zolyno g. 36, 10210 Vilnius, Lithuania; 3 Clinic of Obstetrics & Gynaecology, Faculty of Medicine, Vilnius University, Ciurlionio g. 21/27, 03101 Vilnius, Lithuania; 4 Centre of Obstetrics & Gynaecology, Vilnius University Hospital Santaros Klinikos, Santariskiu g. 2, 08661 Vilnius, Lithuania; 5 Antiaging Clinic, Sakalu g. 22, 08108 Vilnius, Lithuania; 6 Ona Gureviciene Family Clinic, Gedimino g. 17, 68307 Marijampole, Lithuania; 7 Departament of Gynaecology, Vilnius City Clinical Hospital, Antakalnio g. 57, 10207 Vilnius, Lithuania

**Keywords:** *Gardnerella vaginalis*, Bacterial vaginosis, Clade, Subgroup, Sialidase A, Vaginal microbiota

## Abstract

**Background:**

Bacterial vaginosis (BV) is one of the leading causes of vaginal complaints among women of childbearing age. The role of *Gardnerella vaginalis* remains controversial due to its presence in healthy and BV-type vaginal microflora. The phenotypic and genotypic heterogeneity of *G. vaginalis* suggested the existence of strain variants linked with different health conditions. We sought to analyze prevalence and distribution of *G. vaginalis* subgroups (clades) in BV-positive (*n* = 29), partial BV (*n* = 27), and BV-negative (*n* = 53) vaginal samples from Lithuanian women.

**Methods:**

Vaginal samples were characterized by Amsel criteria and the Nugent method. Bacterial signatures characteristic of BV and concomitant infections were identified by culture and PCR. Using singleplex PCR assays, *G. vaginalis* subgroups were identified in 109 noncultured vaginal specimens by targeting clade-specific genes. Isolated *G. vaginalis* clinical strains were subtyped and the presence of the sialidase coding gene was detected by PCR. Data analysis was performed using GraphPad Prism statistical software.

**Results:**

*G. vaginalis* was found in 87% of women without BV. Clade 4 was most frequently detected (79.4%), followed by clade 1 (63.7%), clade 2 (42.2%), and clade 3 (15.7%). Multi-clade *G. vaginalis* communities showed a positive association with Nugent score (NS) ≥ 4 (OR 3.64; 95% CI 1.48–8.91; *p* = 0.005). Clade 1 and clade 2 were statistically significantly more common in samples with NS 7–10 (OR 4.69; 95% CI 1.38–15.88; *p* = 0.01 and OR 6.26; 95% CI 2.20–17.81; *p* ≤ 0.001, respectively). Clade 3 and clade 4 showed no association with high NS (OR 0.88; 95% CI 0.26–3.04; *p* = 1.00 and OR 1.31; 95% CI 0.39–4.41; *p* = 0.767, respectively). The gene coding for sialidase was detected in all isolates of clade 1 and clade 2, but not in clade 4 isolates.

**Conclusions:**

We showed an association between the microbial state of vaginal microflora and specific subgroups of *G. vaginalis*, the distribution of which may determine the clinical manifestation of BV. The frequent detection of clade 4 in the BV-negative samples might be due its lack of the gene coding for sialidase.

**Electronic supplementary material:**

The online version of this article (doi:10.1186/s12879-017-2501-y) contains supplementary material, which is available to authorized users.

## Background

A shift in the vaginal microflora from a Lactobacillus-dominated environment to a heterogeneous community of anaerobic and/or aerobic bacteria is associated with health issues [1]. This depletion of *Lactobacillus* combined with the overgrowth of anaerobic and microaerophilic bacterial species defines bacterial vaginosis (BV) [2, 3]. By causing an abnormal malodorous vaginal discharge, BV has an impact on women’s sexual relationships and quality of life [4]. Moreover, BV is linked with pelvic inflammatory disease [5] and greater susceptibility to sexually transmitted infections [6]. BV-associated bacteria have been related to increased risk of preterm birth [7] and adverse neonatal outcomes [8]. The laboratory method generally accepted as the gold standard for diagnosis of BV is microscopy of Gram-stained vaginal smears [9]. Because both the Nugent scoring system and the Amsel criteria [10] used in clinical practice have their limitations [1, 11], DNA-based assays targeting the bacteria associated with BV are being sought for objective, reproducible, and accurate diagnosis of the BV state [11, 12]. BV is recognized as a polymicrobial disease, but determination of the key pathogenic microbial species and strain variants involved is still ongoing [13–15].

The most studied vaginal anaerobe, *Gardnerella vaginalis*, has been recovered from the vaginal samples of almost all women with BV [3, 11, 12]. *G. vaginalis* possesses a number of virulence factors including production of sialidase A [16, 17] and the toxin vaginolysin [16]. It also is able to adhere to vaginal epithelial cells and establish a biofilm [15, 18]. Although *G. vaginalis* is associated with various clinical conditions, it has been found in vaginal samples of healthy individuals, albeit often in lower numbers than in BV cases [12]. Its well known phenotypic and genetic heterogeneity led to the development of genotyping schemes based on comparison of whole genome sequences and *cpn60* genes [19–22]. These approaches differentiated *G. vaginalis* into four distinct clades (1–4) [19, 20] and four corresponding subgroups (A-D) [21, 22]. qPCR genotyping based on clade-specific genes demonstrated a correlation between BV and particular *G. vaginalis* clades present in 60 vaginal samples of women in the USA [20]. It is not yet clear whether distribution of *G. vaginalis* subgroups (clades) is a universally applicable indicator of vaginal health or disease. Analysis of the virulence potential of each subgroup would reveal distinct pathogenic or commensal subgroups within *Gardnerella*, if present.

In this study, we aimed to identify and subtype four subgroups (clades) of *G. vaginalis* in vaginal samples of Lithuanian women with various clinical conditions, and thereby to demonstrate associations between *G. vaginalis* subgroups and BV.

## Methods

### Selection of patients

Samples were obtained from 116 women who attended private gynecology clinics in Vilnius and Marijampole, Lithuania. The study was approved by the Lithuanian Bioethics Committee (approval no. 158200-13-697-223, 12/11/2013; amendment no. 2, 8/12/2015). Written informed consent was obtained from all study participants prior to enrollment. All were Caucasian Lithuanians >18 years of age (range, 22–53 years; mean, 30.8 years). All had come to the clinic for routine gynaecological examination or with self-reported complaints of vaginal itching/burning sensations or increased and/or malodorous discharge. All participants were asked to complete a questionnaire on the current use of hormonal contraceptives, menstrual cycle, and frequency of vaginal infections. Exclusion criteria included menstruation at the time of enrollment, HIV infection, and antibiotic/antimicrobial treatment within 14 days of sampling. Clinical signs observed by the clinician during examination, as well as symptoms reported by the patients, were recorded.

A total of 121 vaginal samples were collected, one from each of 116 participants and two samples, 3 to 6 months apart, from each of 5 participants. Two samples were excluded from further analysis due to insufficient smear quality for Gram-stain microscopy. Four women were pregnant (from 16 to 34 weeks of gestation). Among the 106 women providing information on their menstrual cycle, 45 (42.5%) were in the follicular phase of the cycle. Three women with reported perimenopausal menstrual cycle irregularities were included. Frequent vaginal infections were reported by 66 women, rare infections by 11 women. For birth control, oral contraceptives were taken by 21 women, IUD coils (type not specified) were used by 3 women, and the NuvaRing was used by 1 woman.

### Examination of vaginal samples, *G. vaginalis* isolation, and gene-specific PCR assays

All samples were subjected to Gram-staining and microscopy to assess their Nugent score (NS) [9]. BV diagnosis was also defined by the clinician and included the mandatory presence of three Amsel criteria (elevated pH, clue cells, and fishy odour discharge) [10, 23]. A sample was considered as BV-positive if NS ranged from 7 to 10 and at least three Amsel criteria were present [23]. A case was categorized as partial BV (NS 4–6) if, irrespective of the Amsel criteria, a mixture of normal (or other) flora with zones of typical BV flora was evident upon microscopic examination [1]. A sample was defined as BV-negative if NS corresponded to normal flora (0–3) and less than three of the mandatory Amsel criteria for BV were present [1, 23].

Additional vaginal samples were collected for cultivation experiments and molecular studies. For culturing, a swab taken near mid-vagina was placed in Amies Charcoal Transport Medium (LP Italiana SPA, Italy) and then transported to the microbiology laboratory within 12 h. There the specimen was cultured on Gardnerella agar (bioMérieux, Marcy l’Etoile, France) at 37 °C in 5% CO_2_ for 48 h. Colonies of *G. vaginalis* were identified as described previously [16]. *G. vaginalis* identification was confirmed by amplification of the 16S rRNA gene [24] and sequencing of the obtained PCR product. For DNA extraction and amplification, a vaginal swab (Eswab, Copan, Italy) was collected and frozen until processed. Then the sample was thawed and one portion was used to detect *Chlamydia trachomatis* (CT), *Neisseria gonorrhoeae* (NG), *Trichomonas vaginalis* (TV), *Mycoplasma hominis* (MH*), Mycoplasma genitalium* (MG), *Ureaplasma urealyticum* (UU), and *Ureaplasma parvum* (UP) in a single real-time PCR reaction using the Anyplex II STI-7 Detection kit according to the manufacturer’s instructions (Seegene, South Korea) [25]. The other portion of the specimen was used to detect 12 bacterial species by conventional PCR as described below.

To test for vaginal colonization by yeast irrespective of clinical signs and symptoms reported by the patient, specimens were cultured on Sabouraud Dextrose Agar (BD, Maryland, US) with chloramphenicol [26]. The presence of four *Candida* species (*C. albicans*, *C. tropicalis*, *C. grabrata,* and *C. parapsilosis*) was assessed using multiplex PCR as described earlier [27].

Where indicated, amplified ribosomal DNA restriction analysis (ARDRA) genotyping using TaqI restriction endonuclease was performed as described previously [16, 24]. The sialidase A coding gene (*sld*) was detected using Sia1F and Sia1R [16], as well as Sia2F, 5′-CACGTGGAACATATGGAAATCG and Sia3R, 5′-TAAATGTCTCTTCCATGTTGGCT, primers.

### Bacterial strains

*G. vaginalis* strains 49145 and 14019 were obtained from the American Type Culture Collection (ATCC). *Bifidobacterium bifidum* was purchased from the Leibniz Institute DSMZ-German Collection of Microorganisms and Cell Cultures (Braunschweig, Germany). *B. bifidum* was cultured anaerobically on RCM agar (Oxoid, UK) at 37 °C for 48 h [28]. *L. iners, L. gasseri, L. crispatus,* and *L. jensenii* strains were isolated from vaginal specimens using MRS agar plates (BD Difco, US) incubated at 37 °C in 5% CO_2_ for 48 h [28]. Identification of *Lactobacillus* species was performed by amplification of the 16S rRNA gene using universal fD1 and rP2 primers [29]. The sequences of the obtained PCR fragment were submitted to the Ribosomal Database Project (RDP) database (https://rdp.cme.msu.edu) for species determination [30]. Genomic DNA from *Streptococcus intermedius, Streptococcus pyogenes*, and *Streptococcus pneumoniae* were a kind gift of Lithuanian National Public Health Surveillance Laboratory, Vilnius, Lithuania.

### DNA extraction and bacterium-specific PCR assays

DNA extraction was performed using the GeneJET Genomic DNA Purification Kit according to the manufacturer‘s recommendations (Thermo Fisher Scientific, Vilnius, Lithuania). The sequences of bacterium-specific PCR primers, annealing temperatures, and amplicon sizes are shown in Additional file 1 [11, 31–34]. The three sets of primers used to detect *G. vaginalis* in vaginal samples are presented in Additional file 2 [11, 35, 36]. All primers were purchased from Metabion International AG, Germany. Amplifications were performed in 25 μL reaction mixtures containing 1X Maxima Hot Start Green PCR master Mix (Thermo Fisher Scientific), 0.4 μM each of forward and reverse primers, and 5 μL of genomic DNA. Amplification reactions included initial denaturation for 5 min at 95 °C, 38 amplification cycles consisting of denaturation for 30 s at 95 °C, annealing for 30 s at 54 to 62 °C, and extension for 45 s at 72 °C. The final extension step was prolonged for 7 min. PCR products were separated on 1.5% agarose gels stained with RedSafe dye (iNtRON Biotechnology, South Korea). PCR assay based on the detection of the *G. vaginalis* toxin vaginolysin (VLY) coding gene was capable of detecting 1–10 molecules of the cloned *vly* gene fragment per reaction.

Human apolipoprotein E (ApoE) gene PCR served as a positive internal control for DNA extraction from each vaginal specimen. Human chromosomal DNA was isolated from a blood specimen obtained from a healthy adult volunteer by venipuncture after their written informed consent had been approved by the Council of the Institute of Biotechnology of Vilnius University (Protocol no. 54 of 20/11/2013). The amplification of the 557 bp fragment of the *apoE* gene was performed using ApoF2, 5′-GCATTGCAGGCAGATAGTGA and ApoR, 5′-CCTGTGTGGAACAAGTTCAAG, primers. No template PCR controls were included in the PCR assays.

To identify *Bifidobacterium* species, the *tuf* gene fragment was amplified with *Bifidobacterium* specific primers BIF-1 and BIF-2 [34] and sequenced. The *tuf* sequences obtained were compared with those available in the GenBank database using BLASTn at NCBI (https://blast.ncbi.nlm.nih.gov/Blast.cgi). PCR of serial dilutions of a cloned *tuf* gene fragment of *B. longum* and *B. bifidum* was capable of detecting ≤100 and ≤20 molecules per reaction, respectively.

### *G. vaginalis* clade-specific PCR assays

Four *G. vaginalis* clades were detected by amplification of the following genes: putative α-L-fucosidase (Gv1-fuc1-S and Gv1-fuc1-AS primers), a hypothetical protein (Gv2-hyp-S and Gv2-hyp-AS primers), thioredoxin (Gv3-thi-S and Gv3-thi-AS primers), and chloride transporter (Gv4-cic-S and Gv4-cic-AS primers) [20]. Identification of clades by conventional PCR was performed using DNA extracted from both the characterized vaginal specimens and clinical *G. vaginalis* isolates. PCR mixtures for clade 1, clade 2, and clade 4 containing DNA extracted from clinical isolates were supplemented with betaine to a final concentration of 0.75–1.0 M. The reaction mixture was subjected to 38 cycles of denaturation at 95 °C for 30 s, primer annealing at 60 °C for 30 s and extension at 72 °C for 30 s. The last cycle included a 7 min extension step. PCR products were separated on 1.7% agarose gels stained with RedSafe dye.

### Data analyses

The prevalence of microorganisms and the distribution of *G. vaginalis* clades in the categorized vaginal samples were reported. Statistical analysis, including sensitivity, specificity, odds ratio (OR) with 95% confidence intervals (CI), and agreement (kappa) between different PCR assays for detection of *G. vaginalis* was performed using GraphPad Prism 6 software for Windows (GraphPad Software Inc., La Jolla, CA, USA). Kappa values (with 95% CI) were classified as follows: < 0.20, poor agreement; 0.21–0.40, fair agreement; 0.41–0.60, moderate agreement; 0.61–0.80, good agreement; 0.81–1.00, excellent agreement [37]. 95% CIs for sensitivity and specificity were calculated by the Clopper-Pearson Exact method [38], while the confidence intervals for the odds ratios were calculated by the Woolf (logit) method [39] available in the statistical software. Differences between groups were evaluated by two-tailed Fisher’s exact test [37] with a significance level of *p* < 0.05 using GraphPad Prism 6.

## Results

### Characterization of vaginal samples

To assess the distribution of *G. vaginalis* subgroups in women with either healthy or disturbed vaginal microflora, the state of every vaginal sample was characterized using both the Nugent method [9] and Amsel criteria [10]. Ten samples were excluded from further analysis due to contradictory classifications by Amsel criteria and Nugent scoring that indicated vaginal flora alterations other than fully developed BV or partial BV [1]. To characterize the vaginal microflora of healthy and BV-affected women, the presence of pertinent bacterial species was detected by PCR assays and of *Candida* by culture, while pathogens associated with seven sexually transmitted infections were detected by qPCR.

**Fig. 1:**
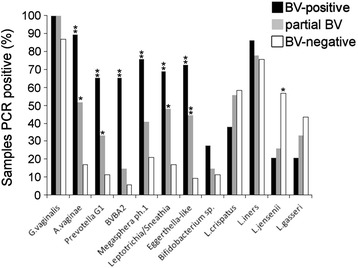
Percentages of vaginal samples positive for bacterial species detected by bacterium-specific PCR *p < 0.05; **p < 0.001

Of the 119 characterized vaginal samples, 29 (24.4%) were classified as BV positive (NS 7–10). Those samples were found by PCR to also be positive for bacteria characteristic of BV (Fig. 1, Additional file 3). In these BV-positive women, the 8 independent qualitative PCR assays yielded averages of 5.7 and 1.7 positive reactions per sample for anaerobic bacteria and for *Lactobacillus* species, respectively. *A. vaginae* and *G. vaginalis* were detected in 89.7% and 100% of BV-positive samples with specificities of 83% (95% CI 70.2–91.9) and 13.2% (95% CI 5.5–25.3), respectively. *Eggerthella*-like (OR 25.20; 95% CI, 7.37–86.19; *p* < 0.001), *Leptotrichia/Sneathia* (OR 10.86; 95% CI 3.75–31.51; *p* < 0.001), and *Megasphera ph.1* (OR 12; 95% CI, 4.08–35.31; *p* < 0.001) species were highly associated with NS 7–10 (Additional file 3). Of the four *Lactobacillus* species, *L. jensenii* was less common in women with BV (OR, 0.2; 95% CI 0.07–0.57). Culture of the 29 analysed BV-positive samples found 8 to be positive for *Candida* (all *C. albicans*). Patients that were positive for both BV and *Candida* colonization reported symptoms of “abnormal vaginal discharge” (8/8) and “vaginal pruritis” (3/8). Overall, 3 of the 29 BV-positive women were asymptomatic.

Fifty-three (44.5%) samples were BV-negative (NS 0–3). In contrast to the BV-positive samples, these BV-negative samples had averages of 1.8 and 2.3 positive reactions per sample for anaerobic bacteria and for *Lactobacillus* species, respectively. In healthy subjects, the most frequently detected bacterial species were *L. iners* (75.5%) and *G. vaginalis* (86.8%), followed by *L. crispatus* (58.5%), *L. jensenii* (56.6%), and *L. gasseri* (43.3%) (Fig. 1, Additional file 3). Among these BV-negative samples, 18.9% (10 of 53) showed yeast colonization. Three of the ten patients who tested positive for yeast by culture and clinician-observed signs of vulvovaginal candidiasis did not report any symptoms, while 7 of the 10 patients reported "increased vaginal discharge and vaginal pruritis".

Of the 119 vaginal samples tested, 27 (22.7%) were designated as partial BV (NS 4–6). In these samples, an average of 3.5 and 1.9 positive reactions were detected per sample for anaerobic bacteria and *Lactobacillus* species, respectively. Here the most common bacterial species were *G. vaginalis* (100%), *L. iners* (77.8%), *L. crispatus* (55.6%), *A. vaginae* (51.9%), and *Leptotrichia/Sneathia* (48.2%). Culturing demonstrated yeast colonization in 2 (7.7%) samples.

**Fig. 2:**
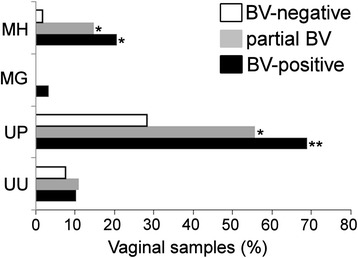
Prevalence of *M. hominis* (MH), *M. genitalium* (MG), *U. parvum* (UP), and *U. urealyticum* (UU) in BV-positive (*n* = 29), partial BV (*n* = 27), and BV-negative (*n* = 53) vaginal samples. *p < 0.05; **p < 0.001

Of the seven pathogens of sexually transmitted infections (STIs) monitored, only the four Mollicutes (*U. urealyticum* [UU]*, U. parvum* [UP], *M. hominis* [MH], and *M. genitalium* [MG]) were found in the analysed vaginal samples using multiplex qPCR [25]. UP was detected in 46% of samples irrespective of the BV status, while MH, UU, and MG were less common (10.1%, 9.2%, and 0.92%, respectively). Co-infection by UP had high association with NS 7–10 (OR 5.63; 95% CI 2.10–15.12; *p* < 0.001) (Additional file 3). Also, the greater prevalence of UP detected in partial BV samples relative to BV-negative samples was statistically significant (OR 3.17; 95% CI 1.21–8.32; *p* = 0.027). Similarly, the increased prevalence of MH in the BV-positive samples compared to the BV-negative samples showed statistical significance (*p* = 0.007). Co-infection by UU was not associated with NS 7–10 (OR 1.41; 95% CI 0.29–6.80; *p* = 0.694) (Fig. 2, Additional file 3).

For subsequent observation of *G. vaginalis* subgroups, the characterized vaginal samples (*n* = 109) were clustered into three categories: BV (*n* = 29), partial BV (*n* = 27), and BV-negative (*n* = 53).

### *G. vaginalis* detection in vaginal samples

The ongoing discussion of *G. vaginalis* prevalence in balanced and unbalanced vaginal flora, as well as the increasing number of PCR assays being used to target BV-associated microorganisms, prompted us to evaluate the specificity of the PCR assays purported to detect *G. vaginalis* in vaginal specimens. These assays used primer pairs that targeted diverse DNA sequences in the *G. vaginalis* genome (Additional file 2): primers GV1 and GV3 target the 23S rRNA gene [35]; primers cpn-For and cpn-Rev target the *cpn60* gene [36]; and primers Gvag 644F and Gvag 851R target the 16S rRNA gene [11]. We designed primers VLY-585F and VLY-1334R (Additional file 1) to amplify a 749 bp fragment of the toxin vaginolysin (VLY) coding gene (*vly*). *vly* sequences determined in our laboratory [16] were aligned and compared with those retrieved from public databases. The VLY coding gene is well-conserved among *G. vaginalis* strains [16]. Its structural and functional homologue, Inerolysin [40], was found only in *Lactobacillus iners*, a common constituent of the vaginal microflora. Specificity of this *vly*-targeting PCR was tested using DNA extracted from 20 *G. vaginalis* strains*, L. iners*, *L. crispatus*, *L. gasseri*, *L. jensenii*, *B. bifidum, S. intermedius, S. pyogenes, S. pneumoniae*, and human chromosomal DNA. No cross-reactivity was detected against non-*G. vaginalis* species, while the *vly* PCR fragment was detected in all *G. vaginalis* isolates tested including species from ATCC. The bacterium specificity of the assay was confirmed by sequencing the PCR product.

A total of 91 vaginal samples (001S1 to 064S1, 093S1 to 0116S1, 005S2, 008S2, and 028S2) were tested for *G. vaginalis* using four sets of primer pairs. Of these samples, 59 (64.8%) tested positive by *cpn60* PCR, 42 (46.2%) by 23S rRNA PCR, 83 (91.2%) by 16S rRNA PCR, and 79 (86.8%) by *vly* PCR. Results for the inter-assay agreement are shown in Additional file 4. The agreement between *vly* PCR and 16S rRNA PCR was moderate (kappa = 0.55), whereas that between *vly* PCR and 23S rRNA PCR was fair (kappa = 0.23). In addition, the *G. vaginalis* subgroups (clades) present were identified in 75 of the 91 (82.4%) vaginal samples by clade-specific PCR assays (see below).

### *G. vaginalis* subgroups in characterized vaginal samples

**Fig. 3:**
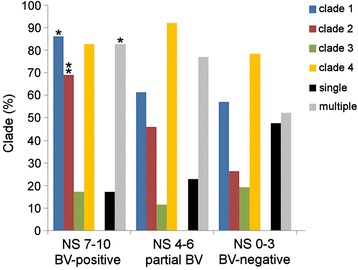
Percentage of *G. vaginalis* clades, as well as single clade and multiple clade communities in BV-positive, partial BV, and BV-negative vaginal samples. *p < 0.05; **p < 0.001

The clade-specific genes that had previously demonstrated the best PCR performance were used as described previously [20] to subtype *G. vaginalis* in the characterized vaginal samples. Of the 102 samples positive for *G. vaginalis*, 5 were negative for all clade-specific PCR tests; no *G. vaginalis* strains were isolated from these specimens. The vast majority of samples contained multiple *G. vaginalis* clades in different combinations (Fig. 3, Additional files 5 and 6). Three clades (clades 1, 3, and 4) were found as single clade communities. Clade 2 was detected only in multi-clade communities, with one exception (sample 093S1; NS 2). Clade 4 was the most frequently detected (79.4%), followed by clade 1 (63.7%), clade 2 (42.2%), and clade 3 (15.7%). Multi-clade *G. vaginalis* communities were positively associated with NS ≥4 (OR 3.64; 95% CI 1.48–8.91; *p* = 0.005).

The prevalence of each of the four *G. vaginalis* clades showed a distinct association with BV status. The least frequently detected clade, clade 3, was almost equally distributed among BV-positive, partial BV, and BV-negative samples (Fig. 3). The most ubiquitous clade, clade 4, showed no association (OR 1.31; 95% CI 0.39–4.41; *p* = 0.767) with BV (NS 7–10), whereas clade 2 was the group most frequently observed in samples from women with both high NS (7–10) and three Amsel criteria (OR 6.26; 95% CI 2.20–17.81; *p* < 0.001). Clade 1 showed a positive association with vaginal flora characterized by NS 7–10 (OR 4.69; 95% CI 1.38–15.88; *p* = 0.010), but was also found in 61.5% and 57.1% of partial BV and BV-negative samples, respectively (Fig. 3, Additional file 5).

### Subtyping of *G. vaginalis* isolates and distribution of the sialidase A coding gene

*G. vaginalis* clinical strains were isolated from 27 characterized vaginal samples without selection based on NS. Each plate was inoculated from a single vaginal swab*. G. vaginalis* was identified as described in Methods. Isolates from individual colonies were then subtyped by clade-specific PCR. Multiple strains of *G. vaginalis* (from 3 to 15) were thus isolated from each swab, specifically 13 isolates per swab for clade 1, 9 for clade 2, 15 for clade 4, and none from clade 3. When classified by BV status, 13 *G. vaginalis* strains were isolated from BV-positive vaginal samples, 8 strains from partial BV samples, and 4 strains from BV-negative samples. The isolates from 9 samples included strains of two different clades. Overall, the clade assignments of these isolated strains matched the clade(s) identified in that sample by PCR, with two exceptions: i) clade 1 was detected in the noncultured vaginal sample 070S1, but the four isolates originated from the corresponding vaginal swab were clade 2; ii) one of seven *G. vaginalis* isolates from sample 058S2 was clade 2 – a clade not detected in the corresponding noncultured vaginal sample.

*G. vaginalis* isolates assigned to all three clades were tested for the presence of the sialidase A coding gene (*sld*) using two primer pairs (see Methods). The 35 clade 4 strains tested that had been derived from 15 samples with varied NS were all negative for *sld*, whereas *G. vaginalis* ATCC 14019 and strains of clade 1 (*n* = 13) and clade 2 (*n* = 12) were positive.

### *G. vaginalis* isolates of an unknown clade

Some of the strains isolated from samples 060S1, 086S1, 099S1, and 108S1 did not belong to any clade detectable by clade-specific PCR, although all of them were identified as *G. vaginalis* by both their characteristic microbiological profile and the nucleotide sequence of their 16S rRNA coding gene (RDP, https://rdp.cme.msu.edu, [30]). Clade-specific PCR identified four clades in vaginal sample 099S1 (Additional file 6), but found none from known clades in three *G. vaginalis* isolates from the corresponding swab (Table 1). Sample 060S1 (NS 10; clade 2 + clade 4) yielded 15 independent isolates: 11 from clade 2 and 4 that did not belong to any of the four known clades. Vaginal sample 086S1 (NS 6) harboured all four clades. The corresponding swab yielded 3 isolates from clade 2 and 7 that belonged to a subgroup unidentifiable by clade-specific PCR. The 7 isolates from vaginal sample 086S1 assigned to the unidentified clade were tested by PCR targeting the genes coding for the virulence factors vaginolysin (*vly*) and sialidase A (*sld*). All 7 isolates tested negative for *vly*, but found positive for *sld* (Table 1).

**Table 1:** Characteristics of *G. vaginalis* clinical isolates of the unknown clade

Sample no.	Number of isolates isolates strains	ARDRA genotype (TaqI)	Gene	
			*vly*	*sld*
060S1	4	2	+	*+*
086S1	7	1	−	*+*
099S1	3	1	+	*+*
108S1	1	3	+	−

All isolates of the unknown clade exhibited the *Gardnerella*-specific ARDRA genotype (Table 1). It remains to be determined whether these isolates represent one or more new clades or are derivatives of known clades that have undergone genetic rearrangements or mutations in clade-specific genes.

## Discussion

The role of *Gardnerella vaginalis* in vaginal disease remains controversial due to its presence in both healthy and BV-type vaginal microflora. To shed further light on this question, in this study we first characterized vaginal samples by Amsel criteria and the Nugent method to discriminate between normal vaginal flora and the BV condition. Because vaginal co-infections and mixed infections may mimic or mask recognition of BV, we tested these samples for *Candida* by culture [23] and for seven STIs by qPCR assays. Previous studies [3, 11, 12] had demonstrated that women with clinically diagnosed BV were carrying multiple anaerobic bacteria, while only a minority of BV-negative subjects tested positive for more than two BV-related bacteria. Here we found multiple *Lactobacillus* species in vaginal samples from the majority of BV-negative women. In contrast, *L. iners*, alone or in combination with *L. crispatus*, was most frequently detected in the BV-positive and partial BV samples. Among *Lactobacillus* species, only *L. jensenii* was inversely associated with NS ≥ 4; *L. crispatus* was not [11]. Five bacterial species, namely *A. vaginae*, *G. vaginalis*, *Eggerthella*-like, *Megasphera ph. 1*, and *Leptotrichia/Sneathia*, were found in most subjects with BV and thus can be considered to be bacterial indicators of this disorder. qPCR targeting the *tuf* gene identified two *Bifidobacterium* species, *B. bifidum* and *B. longum*, in the vaginal samples. The frequency of colonization (or co-infection) of *Candida* and BV-associated bacterial species was consistent with the published data on co-infections and mixed vaginal infections [41]. However, symptoms and clinical signs were indicative of contributions from two pathogens in the minority of BV-affected women.

Testing for the incidence of concomitant colonization by several important human pathogens revealed possible synergy with BV. Specifically, UP and MH were more prevalent in women with BV than in women with non-BV type flora, while UU showed no such association. This greater prevalence of particular genital Mollicutes in BV-positive samples cannot be attributed solely to co-infection by *G. vaginalis* [42] because the latter was also common in the BV-negative samples. However, quantification of microbial loads in the vaginal samples might reveal a synergistic relationship between BV-associated bacteria and vaginal Mollicutes [42].

*G. vaginalis* was found to be prevalent in women without BV, an observation in agreement with data from several patient cohorts in the USA [11, 20]. Likewise, the lack of specificity of *cpn60* PCR as a test for *G. vaginalis* observed agrees with an earlier report [20]. However, we did not find an association between false-negative results from *cpn60* PCR or 23S rRNA PCR and particular *G. vaginalis* clades, either individually or in combination. The differences in *G. vaginalis* detection observed for various PCR primers demonstrate that the low prevalence of *G. vaginalis* reported in BV-negative women might depend not only on the patient’s cohorts or geographical/ethnic origin, but also on the target sequences selected for PCR.

Subtyping of the *G. vaginalis* present in noncultured vaginal samples by qualitative clade-specific PCR [20] demonstrated a significant association between multi-clade *G. vaginalis* communities and NS ≥ 4. This agrees with the findings of a recent study [20] which suggested that the presence of multiple clades was a result of unprotected sex with new partners. Indeed, the prevalence of polyclonal *G. vaginalis* communities in the vaginal milieu once again raises the question of the mode of BV acquisition [43].

Within the vaginal samples with single clade communities (*n* = 31), clade 4 (*n* = 19) and clade 1 (*n* = 9) were most prevalent regardless of the BV status. The less often detected clade 3 demonstrated no association with the disorder. An earlier study in the USA detected clade 3 in 31.7% of the 60 samples studied and showed its association with BV-type flora [20]. While we found no clade 3 *G. vaginalis* strains among the isolates, strains of subgroup D corresponding to clade 3 were isolated from specimens that originated from Canada (*n* = 1) and Kenya (*n* = 7) [22]. Clade 4, the most frequently found clade, showed no association with BV, an observation that is in agreement with previous results [20]. Our finding that *G. vaginalis* strains of clade 1 were more likely to colonize BV-positive women (*p* = 0.010) was consistent with the previous finding by Balashov and colleagues [20]. Although we found that clade 2 was significantly more common in the samples with high Nugent scores (7–10), that clade had been reported to be associated with intermediate vaginal flora (NS 4–6) in samples from the USA [20]. The observed discrepancies might reflect the small number of clinical samples analysed in both of these studies, or, because one study was performed in the USA and one in Lithuania, the ethnicity and geographical location of patients may have also been a factor. In addition, the two studies employed different PCR techniques: singleplex, conventional, clade-specific PCR (this study) and multiplex qPCR for the samples from the USA [20].

The presence of *sld* does not necessarily indicate expression and/or activity of sialidase A [16, 17, 22]. However, clade 4 strains characteristically lack the sialidase A coding gene. Strains of the corresponding *cpn60* subgroup A were also negative for the *sld* gene [22]. Because sialidase A can degrade the mucous layer [17], its absence might explain the high detection frequency of *G. vaginalis* clade 4 in normal vaginal flora. It is not clear whether clade 4 strains produce other mucinases whose activity may affect clinical status.

Isolation and subtyping of *G. vaginalis* strains confirmed the subgroup assignments from the noncultured vaginal samples. However, the results of clade-specific PCR of isolated *G. vaginalis* strains from four vaginal samples did not match any known type, even though they demonstrated a specific genetic profile (Table 1). Combined, these results suggest the possible existence of more than four *G. vaginalis* subgroups.

## Conclusions

This study analysed the relevance of *G. vaginalis* genotyping approach based on the detection of clade-specific genes to various clinical conditions in the vagina. *G. vaginalis* was highly prevalent in BV-negative Lithuanian women. Subtyping of *G. vaginalis* in vaginal samples demonstrated that multi-clade *G. vaginalis* communities were more common in vaginal samples with NS ≥ 4. Detection of clade 1 and clade 2 was associated with NS 7–10, but there was no association of either clade 3 or clade 4 with high Nugent scores. These results were confirmed by subtyping *G. vaginalis* clinical isolates. The isolation of *G. vaginalis* strains of an unknown subtype underscores the high complexity of the genus *Gardnerella* that still remains to be investigated. The gene for sialidase A was detected in all isolates of clade 1 and clade 2, but not in clade 4 isolates. Our study demonstrates that the high prevalence of *G. vaginalis* detected in women without BV does not disprove the hypothesis that *G. vaginalis* is a causative agent of BV because the functional role played by *G. vaginalis* within the vaginal microflora could differ significantly depending on the particular subgroup(s) dominating the BV-type flora. Future studies will focus on elucidating the clinically significant phenotypic properties of the various *G. vaginalis* subgroups.

## Additional files

Additional file 1: Bacterial strains, primer sequences and PCR conditions for bacterium-specific PCR assays. (PDF 231 kb)

Additional file 2: Primers and PCR conditions for *Gardnerella vaginalis* detection by PCR assay. (PDF 216 kb)

Additional file 3: Data analysis comparing the detection frequency of microorganisms by PCR assays in BV-positive, partial BV, and BV-negative samples. (PDF 171 kb)

Additional file 4: Agreement between various gene-specific PCR tests employed to detect *G. vaginalis* in vaginal samples. (PDF 146 kb)

Additional file 5: Data analysis comparing the detection frequency of four *G. vaginalis* clades by clade-specific PCR assays in BV-positive, partial BV, and BV-negative samples. (PDF 158 kb)

Additional file 6: Characterization of 109 vaginal samples by Nugent score, *vly* PCR for *G. vaginalis*, and clade-specific PCR. (PDF 142 kb)

## Abbreviations

ARDRA: amplified ribosomal DNA restriction analysis
BV: bacterial vaginosis
CT: *Chlamydia trachomatis*
MG: *Mycoplasma genitalium*
MH: *Mycoplasma hominis*
NG: *Neisseria gonorrhoeae*
NS: Nugent score
RDP: Ribosomal Database Project
STIs: sexually transmitted infections
TV: *Trichomonas vaginalis*
UP: *Ureaplasma parvum*
UU: *Ureaplasma urealyticum*

## References

[1] Donders GG (2007). Definition and classification of abnormal vaginal flora. Best Pract Res Clin Obstet Gynaecol.

[2] Srinivasan S, Fredricks DN (2008). The human vaginal bacterial biota and bacterial vaginosis. Interdiscip Perspect Infect Dis.

[3] Srinivasan S, Hoffman NG, Morgan MT, Matsen FA, Fiedler TL, Hall RW (2012). Bacterial communities in women with bacterial vaginosis: high resolution phylogenetic analyses reveal relationships of microbiota to clinical criteria. PLoS One.

[4] Bilardi JE, Walker S, Temple-Smith M, McNair R, Mooney-Somers J, Bellhouse C (2013). The burden of bacterial vaginosis: women‘s experience of the physical, emotional, sexual and social impact of living with recurrent bacterial vaginosis. PLoS One.

[5] Haggerty CL, Totten PA, Tang G, Astete SG, Ferris MJ, Norori J (2016). Identification of novel microbes associated with pelvic inflammatory disease and infertility. Sex Transm Infect.

[6] Allsworth JE, Peipert JF**.** Severity of bacterial vaginosis and the risk of sexually transmitted infection. Am J Obstet Gynecol. 2011;205:113.e1–6. 10.1016/j.ajog.2011.02.060 PMC3156883 21514555

[7] Nelson DB, Hanlon A, Nachamkin I, Haggerty C, Mastrogiannis DS, Liu C (2014). Early pregnancy changes in bacterial vaginosis-associated bacteria and preterm delivery. Paediatr Perinat Epidemiol.

[8] Dingens AS, Fairfortune TS, Reed S, Mitchell C (2016). Bacterial vaginosis and adverse outcomes among full-term infants: a cohort study. BMC Pregnancy Childbirth.

[9] Nugent RP, Krohn MA, Hillier SL (1991). Reliability of diagnosing bacterial vaginosis is improved by a standardized method of gram stain interpretation. J Clin Microbiol.

[10] Amsel R, Totten PA, Spiegel CA, Chen KC, Eschenbach D, Holmes KK (1983). Nonspecific vaginitis. Diagnostic criteria and microbial and epidemiologic associations. Am J Med.

[11] Fredricks DN, Fiedler TL, Thomas K, Oakley BB, Marrazzo JM (2007). Targeted PCR for detection of vaginal bacteria associated with bacterial vaginosis. J Clin Microbiol.

[12] Zozaya-Hinchliffe M, Lillis R, Martin DH, Ferris MJ (2010). Quantitative PCR assessments of bacterial species in women with and without bacterial vaginosis. J Clin Microbiol.

[13] Schwebke JR, Muzny CA, Josey WE. Role of *Gardnerella vaginalis* in the pathogenesis of bacterial vaginosis: a conceptual model. J Infect Dis. 2014;210:338–43. 10.1093/infdis/jiu089 24511102

[14] Srinivasan S, Munch MM, Sizova MV, Fiedler TL, Kohler CM, Hoffman NG, et al. More easily cultivated than identified: classical isolation with molecular identification of vaginal bacteria. J Infect Dis. 2016;214(Suppl S1):S21–28. 10.1093/infdis/jiw192 PMC4957512 27449870

[15] Patterson JL, Stull-Lane A, Girerd PH, Jefferson KK (2010). Analysis of adherence, biofilm formation and cytotoxicity suggests a greater virulence potential of *Gardnerella vaginalis* relative to other bacterial vaginosis-associated anaerobes. Microbiology.

[16] Pleckaityte M, Janulaitiene M, Lasickiene R, Zvirbliene A (2012). Genetic and biochemical diversity of *Gardnerella vaginalis* strains isolated from women with bacterial vaginosis. FEMS Immunol Med Microbiol.

[17] Lewis WG, Robinson LS, Perry JC, Lewis AL (2013). Degradation, foraging, and depletion of mucus sialoglycans by the vagina-adapted *Actinobacterium Gardnerella vaginalis*. J Biol Chem.

[18] Castro J, Alves P, Sousa C, Cereija T, França Â, Jefferson KK (2015). Using an *in-vitro* biofilm model to assess the virulence potential of bacterial vaginosis or non-bacterial vaginosis *Gardnerella vaginalis* isolates. Sci Rep.

[19] Ahmed A, Earl J, Retchless A, Hillier SL, Rabe LK, Cherpes TL (2012). Comparative genomic analyses of 17 clinical isolates of *Gardnerella vaginalis* provide evidence of multiple genetically isolated clades consistent with subspeciation into genovars. J Bacteriol.

[20] Balashov SV, Mordechai E, Adelson ME, Gygax SE (2014). Identification, quantification and subtyping of *Gardnerella vaginalis* in noncultured clinical vaginal samples by quantitative PCR. J Med Microbiol.

[21] Paramel Jayaprakash T, Schellenberg JJ, Hill JE (2012). Resolution and characterization of distinct *cpn60*-based subgroups of *Gardnerella vaginalis* in the vaginal microbiota. PLoS One.

[22] Schellenberg JJ, Paramel Jayaprakash T, Withana Gamage N, Patterson MH, Vaneechoutte M, Hill JE. *Gardnerella vaginalis* subgroups defined by *cpn60* sequencing and sialidase activity in isolates from Canada, Belgium and Kenya. PLoS One. 2016;11(1):e0146510. 10.1371/journal.pone.0146510 PMC4709144 26751374

[23] CDC (2015). Sexually transmitted diseases treatment guidelines. MMWR Recomm Rep.

[24] Ingianni A, Petruzzelli S, Morandotti G, Pompei R (1997). Genotypic differentiation of *Gardnerella vaginalis* by amplified ribosomal DNA restriction analysis (ARDRA). FEMS Immunol Med Microbiol.

[25] Choe HS, Lee DS, Lee SJ, Hong S, Park DC, Lee MK, et al. Performance of Anyplex™ II multiplex real-time PCR for the diagnosis of seven sexually transmitted infections: comparison with currently available methods. Int J Inf Dis. 2013;17(12):e1134–40. 10.1016/j.ijid.2013.07.011 24095619

[26] Pincus DH, Orenga S, Chatellier S (2007). Yeast identification – past, present, and future methods. Med Mycol.

[27] Luo G, Mitchell TG (2002). Rapid identification of pathogenic fungi directly from cultures using multiplex PCR. J Clin Microbiol.

[28] Hartemink R, Rombouts FM (1999). Comparison of media for the detection of bifidobacteria, lactobacilli and total anaerobes from faecal samples. J Microbiol Methods.

[29] Weisburg WG, Barns SM, Pelletier DA, Lane DJ (1991). 16S ribosomal DNA amplification for phylogenetic study. J Bacteriol.

[30] Cole JR, Wang Q, Fish JA, Chai B, McGarrell DM, Sun Y, et al. Ribosomal Database Project: data and tools for high throughput rRNA analysis. Nucleic Acids Res. 2014;42(D1). 10.1093/nar/gkt1244 PMC3965039 24288368

[31] Verhelst R, Verstraelen H, Claeys, Verschraegen G, Delanghe J, Van Simaney L, et al. Cloning of 16S rRNA genes amplified from normal and disturbed vaginal microflora suggests a strong association between *Atopobium vaginae*, *Gardnerella vaginalis* and bacterial vaginosis. BMC Microbiol. 2004;4:16. 10.1186/1471-2180-4-16 PMC419343 15102329

[32] De Backer E, Verhelst R, Verstraelen H, Alqumber MA, Burton JP, Tagg JR, et al. Quantitative determination by real-time PCR of four vaginal *Lactobacillus* species, *Gardnerella vaginalis* and *Atopobium vaginae* indicates an inverse relationship between *L. gasseri* and *L. iners*. BMC Microbiol. 2007;7:115. 10.1186/1471-2180-7-115 PMC2233628 18093311

[33] Byun R, Nadkarni MA, Chhour KL, Martin FE, Jacques NA, Hunter N (2004). Quantitative analysis of diverse *Lactobacillus* species present in advanced dental caries. J Clin Microbiol.

[34] Ventura M, Canchaya C, Meyland V, Klaenhammer TR, Zink R (2003). Analysis, characterization, and loci of the *tuf* genes in *Lactobacillus* and *Bifidobacterium* species and their direct application for species identification. Appl Environ Microbiol.

[35] Zariffard MR, Saiffudin M, Sha BE, Spear GT. Detection of bacterial vaginosis-related organisms by real-time PCR for Lactobacilli, *Gardnerella vaginalis* and *Mycoplasma hominis*. FEMS Immunol Med Microbiol. 2002;34:277–81. 10.1111/j.1574-695X.2002.tb00634.x 12443827

[36] Menard JP (2008). Fenollar F, Henry M, Bretelle F, Raoult. Molecular quantification of *Gardnerella vaginalis* and *Atopobium vaginae* loads to predict bacterial vaginosis. Clin Infect Dis.

[37] Fletcher RH, Fletcher SW (2005). Clinical epidemiology: the essentials.

[38] Shan G. Exact confidence limits for the response rate in two-stage designs with over- or under-enrollment in the second stage. Stat Methods Med Res. 2016; pii: 0962280216650918. 10.1177/0962280216650918 PMC5219879 27389669

[39] Agresti A (1999). On logit confidence intervals for the odds ratio with small samples. Biometrics.

[40] Rampersaud R, Planet PJ, Randis TM, Kulkarni R, Aguilar JL, Lehrer RI (2011). Inerolysin, a cholesterol-dependent cytolysin produced by *Lactobacillus iners*. J Bacteriol.

[41] Rivers CA, Adaramola OO, Schwebke JR (2011). Prevalence of bacterial vaginosis and vulvovaginal candidiasis mixed infection in a southeastern american STD clinic. Sex Transm Dis.

[42] Cox C, Watt AP, McKenna JP, Coyle PV (2016). *Mycoplasma hominis* and *Gardnerella vaginalis* display a significant synergistic relationship in bacterial vaginosis. Eur J Clin Microbiol Infect Dis.

[43] Verstraelen H (2008). Bacterial vaginosis: a sexually enhanced disease. Int J STD AIDS.

