# Supplementary material for: Prevalence and distribution of Gardnerella vaginalis subgroups in women with and without bacterial vaginosis
Source: BMC Infect Dis. 2017 Jun 5;17:394. doi: 10.1186/s12879-017-2501-y (PMC5460423; doi:10.1186/s12879-017-2501-y)
Supplement: Supplementary file 1 — Bacterial strains, primer sequences and PCR conditions for bacterium-specific PCR assays. (PDF 231 kb) [file 12879_2017_2501_MOESM1_ESM.pdf]

Additional file 1. Bacterial strains, primer sequences and PCR conditions for bacterium-specific PCR assays

Specificity	Primer	Primer sequence (5'-3')	Reference	Annealing temp. (°C)	Amplicon (bp)
Lactobacillus crispatus	Lcrisp-452F	GATAGAGGTAGTAACTGGCCTTTA	11	54	571
	Lcrisp-1023R	CTTTGTATCTCTACAAATGGCACTA			
Prevotella G1	PrevG1-468R	GTCCCTTATTGCATGTACCATAC	11	55	389
	PrevG1-857R	GCCGCTAACACTAGGTGCTA			
Lactobacillus inners	Liners-452L	ACAGGGGTAGTAACTGACCTTTG	11	55	569
	Liners-1022R	ATCTAATCTCTTAGACTGGCTATG			
Megasphera phylotype 1	MegaE-456F	GATGCCAACAGTATCCGTCCG	11	55	211
	MegaE-667R	CCTCTCCGACACTCAAGTTCGA			
Leptotrichia/Sneathia spp.	Lepto-359F	CAATTCTGTGTGTGTGAAGAAG	11	55	287
	Lepto-646R	ACAGTTTTGTAGGCAAGCCTAT			
Eggerthella-like bacterium	Egger-621F	AACCTCGAGCCGGGTTCC	11	55	238
	Egger-859R	TCGGCACGGAAGATGTAATCT			
BVBA2	BVBA2-619F	TTAACCTTGGGGTTCATTACAA	11	55	405
	BVBA2-1024R	AATTCAGTCTCCTGAATCGTCAGA			
Atopobium vaginae	Ato-154F	ATATTTGTCGCATGGCGAAT	31	58	433
	Ato-587R	GAGCGGATAGGGGTTGAGC			
Lactobacillus jensenii	JenTR2F	CCTTAAGTCTGGGATACCATT	32	58	90
	JenTR2R	ACGCCGCCTTTTAAACTTCTT			
Lactobacillus gasseri	LactoF	TGGAAACAGRTGCTAATACCG	33	60	322
	LgassR	CAGTTACTACCTCTATCTTTCTTCACTAC			
Gardnerella vaginalis	VLY-585F	GTACGATTCTGCAAGCGCACAAAGC	this study	60	749
	VLY-1334R	CCTTCCCAAGCGCGAGAACGC			
Bifidobacterium sp.	Bif.tuf-1	GAGTACGACTTCAACCAG	34	60	1000
	Bif.tuf-2	CAGGCGAGGATCTTGGT			
