# Supplementary material for: Prevalence and distribution of Gardnerella vaginalis subgroups in women with and without bacterial vaginosis
Source: BMC Infect Dis. 2017 Jun 5;17:394. doi: 10.1186/s12879-017-2501-y (PMC5460423; doi:10.1186/s12879-017-2501-y)
Supplement: Supplementary file 2 — Primers and PCR conditions for Gardnerella vaginalis detection by PCR assay. (PDF 216 kb) [file 12879_2017_2501_MOESM2_ESM.pdf]

Additional file 2. Primers and PCR conditions for *Gardnerella vaginalis* detection by PCR assay

**Primer**	**Primer sequence (5'-3')**	**Reference**	**Annealing temp. (°C)**	**Amplicon (bp)**
GV1	TTACTGGTGTATCACTGTAAGG	35	55	334
GV3	CCGTCACAGGCTGAACAGT			
cpn-For	CGCATCTGCTAAGGATGTTG	36	58	72
cpn-Rev	CCAGGCATGTAAGCCCAAA			
Gvag-644F	GGGCGGGCTAGAGTGCA	11	62	207
Gvag-851R	GAACCCGTGGAATGGGCC			
