# Supplementary material for: Prevalence and distribution of Gardnerella vaginalis subgroups in women with and without bacterial vaginosis
Source: BMC Infect Dis. 2017 Jun 5;17:394. doi: 10.1186/s12879-017-2501-y (PMC5460423; doi:10.1186/s12879-017-2501-y)
Supplement: Supplementary file 3 — Data analysis comparing the detection frequency of microorganisms by PCR assays in BV-positive, partial BV, and BV-negative samples. (PDF 171 kb) [file 12879_2017_2501_MOESM3_ESM.pdf]

Additional file 3. Data analysis comparing the detection frequency of microorganisms by PCR assays in BV-positive, partial BV and BV-negative samples.

Microorganism	Partial BV vs BV-negative				BV-positive vs BV-negative			
	**Sensitivity (%, 95% CI)**	**Specificity (%, 95% CI)**	***p***	**OR (95% CI)**	**Sensitivity (%, 95% CI)**	**Specificity (%, 95% CI)**	***p***	**OR (95% CI)**
Gardnerella vaginalis	100 (87.2-100)	13.2 (5.5-25.3)	0.088	8.87 (0.49-61.54)	100 (88.1-100)	13.2 (5.5-25.3)	0.048	9.52 (0.52-173.01)
Atopobium vaginae	51.9 (32.0-71.4)	83.0 (70.2-91.9)	0.002	5.27 (1.86-14.92)	89.7 (72.6-97.8)	83.0 (70.2-91.9)	<0.001	42.37 (10.51-170.80)
Prevotella G1	33.3 (16.5-54.0)	88.7 (77.0-95.7)	0.031	3.917 (1.22-12.59)	65.5 (45.6-82.1)	88.7 (76.9-95.7)	<0.001	14.88 (4.74-46.73)
BVAB2	14.8 (4.2-33.7)	94.3 (84.3-98.8)	0.218	2.90 (0.60-14.03)	65.5 (45.6-82.1)	94.3 (84.3-98.8)	<0.001	31.67 (7.85-127.70)
Megasphera phylotype 1	40.7 (22.4-61.2)	79.2 (65.9-89.2)	0.069	2.62 (0.95-7.24)	75.8 (56.5-89.7)	79.2 (65.9-89.2)	<0.001	12.00 (4.08-35.31)
Leptotrichia/Sneathia spp.	48.2 (28.6- 68.0)	83.0 (70.2-91.9)	0.007	4.54 (1.60-12.86)	68.9 (49.2-84.7)	83.0 (70.2-91.9)	<0.001	10.86 (3.75-31.51)
Eggerthella-like bacteria	44.4 (25.5-64.7)	90.6 (79.4-96.8)	<0.001	7.68 (2.33-25.34)	72.4 (52.7-87.2)	90.6 (79.4-96.9)	<0.001	25.20 (7.37-86.19)
L.crispatus	55.6 (35.3-74.5)	41.5 (28.2-55.9)	0.816	0.88 (0.35-2.26)	37.9 (20.7-57.8)	41.5 (28.1-55.9)	0.106	0.43 (0.17-1.09)
L.inners	77.8 (57.8-91.4)	24.5 (13.7-38.2)	1.000	1.14 (0.38-3.42)	86.2 (68.3-96.1)	24.5 (13.8-38.2)	0.393	2.03 (0.59-6.93)
L.jensenii	25.9 (11.1-46.3)	43.4 (29.8-57.7)	0.010	0.27 (0.10-0.74)	20.7 (8.0-39.8)	43.4 (29.8-57.7)	0.002	0.20 (0.07-0.57)
L.gasseri	33.3 (16.5-54.0)	56.6 (42.3-70.2)	0.472	0.65 (0.25-1.72)	20.7 (8.0-39.8)	56.6 (42.3-70.2)	0.054	0.34 (0.12-0.97)
Bifidobacterium sp.	14.8 (4.2-33.7)	88.7 (77.0-95.7)	0.726	1.36 (0.35-5.31)	27.6 (12.8-47.2)	88.7 (76.9-95.7)	0.073	2.98 (0.92-9.68)
U. urealiticum (UU)	11.1 (2.4-29.2)	92.4 (81.8-97.9)	0.683	1.53 (0.32-7.40)	10.3 (2.2-27.4)	92.4 (81.8-97.9)	0.694	1.41 (0.29-6.80)
U. parvum (UP)	55.6 (35.3-74.5)	71.7 (57.6-83.2)	0.027	3.17 (1.21-8.32)	69.0 (49.2-84.7)	71.7 (57.6-83.2)	<0.001	5.63 (2.10-15.12)
M. genitalium (MG)	-	-	-	-	-	-	-	-
M. hominis (MH)	14.8 (4.2-33.7)	98.1 (90.0-99.9)	0.042	9.04 (0.96-85.48)	20.7 (8.0-39.8)	98.1 (89.9-99.9)	0.007	13.56 (1.54-119.2)
N.gonorrhoeae (NG)	-	-	-	-	-	-	-	-
Chl. trachomatis (CT)	-	-	-	-	-	-	-	-
T. vaginalis (TV)	-	-	-	-	-	-	-	-
Candida by culture	7.7 (0.9-25.1)	81.1 (68.0-90.6)	0.318	0.36 (0.07-1.77)	27.6 (12.8-47.2)	81.1 (68.0-90.6)	0.409	1.64 (0.56-4.76)
Candida by PCR	30.8 (14.3-51.8)	77.4 (63.8-87.7)	0.583	1.52 (0.53-4.35)	37.9 (20.7-57.8)	81.1 (68.0-90.6)	0.069	2.63 (0.95-7.27)
