# Supplementary material for: Prevalence and distribution of Gardnerella vaginalis subgroups in women with and without bacterial vaginosis
Source: BMC Infect Dis. 2017 Jun 5;17:394. doi: 10.1186/s12879-017-2501-y (PMC5460423; doi:10.1186/s12879-017-2501-y)
Supplement: Supplementary file 4 — Agreement between various gene-specific PCR tests employed to detect G. vaginalis in vaginal samples. (PDF 146 kb) [file 12879_2017_2501_MOESM4_ESM.pdf]

Additional file 4. Agreement between variuos gene-specific PCR tests employed to detect *G.vaginalis* in vaginal samples

PCR assays	Kappa value (interpretation)	
vly PCR vs16S rRNA PCR	Kappa=0.55<br>(moderate)	Number of observed agreements: 83 (91.21% of the observations)<br>Number of agreements expected by chance: 73.1 (80.34% of the observations)<br>Standard error (SE) of kappa=0.14<br>95% CI: 0.28-0.87
vly PCR vs *cpn60* PCR	Kappa=0.44<br>(moderate)	Number of observed agreements: 71 (78.02% of the observations)<br>Number of agreements expected by chance: 55.4 (60.92% of the observations)<br>SE of kappa = 0.09<br>95% CI: 0.26-0.62
vly PCR vs 23S rRNA PCR	Kappa=0.23<br>(fair)	Number of observed agreements: 54 (59.34% of the observations)<br>Number of agreements expected by chance: 42.9 (47.17% of the observations)<br>SE of kappa = 0.06<br>95%CI: 0.11-0.35
16S rRNA PCR vs 23S rRNA PCR	Kappa=0.15<br>(poor)	Number of observed agreements: 50 (54.95% of the observations)<br>Number of agreements expected by chance: 42.6 (46.83% of the observations)<br>SE of kappa = 0.05<br>95%CI: 0.05-0.26
16S rRNA PCR vs *cpn60* PCR	Kappa=0.24<br>(fair)	Number of observed agreements: 65 (71.43% of the observations)<br>Number of agreements expected by chance: 56.6 (62.23% of the observations)<br>SE of kappa = 0.09<br>95% CI: 0.07-0.41
23S rRNA PCR vs *cpn60* PCR	Kappa=0.64<br>(good)	Number of observed agreements: 74 (81.32% of the observations)<br>Number of agreements expected by chance: 44.5 (48.86% of the observations)<br>SE of kappa = 0.07<br>95% CI: 0.49-0.78
