# Supplementary material for: Prevalence and distribution of Gardnerella vaginalis subgroups in women with and without bacterial vaginosis
Source: BMC Infect Dis. 2017 Jun 5;17:394. doi: 10.1186/s12879-017-2501-y (PMC5460423; doi:10.1186/s12879-017-2501-y)
Supplement: Supplementary file 5 — Data analysis comparing the detection frequency of four G. vaginalis clades by clade-specific PCR assays in BV-positive, partial BV, and BV-negative samples. (PDF 158 kb) [file 12879_2017_2501_MOESM5_ESM.pdf]

Additional file 5. Data analysis comparing the detection frequency of four *G. vaginalis* clades by clade-specific PCR assays in BV-positive, partial BV and BV-negative samples.

	**Sensitivity (%, 95% CI)**	**Specificity (%, 95% CI)**	***p***	**OR (95% CI)**
**multiple clades vs single clade**				
BV-positive vs BV-negative	82.7 (64.2-94.2)	47.6 (32.0-63.6)	0.011	4.36 (1.40-13.62)
Partial BV vs BV-negative	76.9 (56.3-91.0)	47.6 (32.0-63.6)	0.071	3.03 (1.013-9.06)
BV-positive+partial BV vs BV-negative	80.0 (67.1-89.6)	47.6 (32.0-63.6)	0.005	3.64 (1.48-8.91)
**clade 1 vs any other clade**				
BV-positive vs BV-negative	86.2 (68.3-96.1)	42.9 (27.7-59.1)	0.010	4.69 (1.38-15.88)
Partial BV vs BV-negative	61.5 (40.6-79.8)	42.9 (27.7-59.1)	0.803	1.2 (0.44-3.26)
BV-positive+partial BV vs BV-negative	74.6 (61.0-85.4)	42.9 (27.7-59.1)	0.084	2.20 (0.93-5.20)
**clade 2 vs any other clade**				
BV-positive vs BV-negative	69.0 (49.2-84.7)	73.8 (58.0-86.1)	<0.001	6.26 (2.20-17.81)
Partial BV vs BV-negative	46.1 (26.6-66.7)	73.8 (58.0-86.1)	0.117	2.42 (0.86-6.79)
BV-positive+partial BV vs BV-negative	58.2 (44.1-71.4)	73.8 (58.0-86.1)	0.002	3.92 (1.64-9.38)
**clade 3 vs any other clade**				
BV-positive vs BV-negative	17.2 (5.8-35.8)	80.9 (65.9-91.4)	1.00	0.88 (0.26-3.04)
Partial BV vs BV-negative	11.5 (2.4-30.1)	80.9 (65.9-91.4)	0.512	0.55 (0.13-2.31)
BV-positive+partial BV vs BV-negative	14.5 (6.5-26.7)	80.9 (65.9-91.4)	0.590	0.72 (0.25-2.12)
**clade 4 vs any other clade**				
BV-positive vs BV-negative	82.8 (64,2-94,2)	21.4 (10.3-36.8)	0.767	1.31 (0.39-4.41)
Partial BV vs BV-negative	92.3 (74.9-99.0)	21.4 (10.3-36.8)	0.184	3.27 (0.65-16.54)
BV-positive+partial BV vs BV-negative	87.3 (75.6-947)	21.4 (10.3-36.8)	0.281	1.87 (0.63-5.52)
