# Supplementary material for: Prevalence and distribution of Gardnerella vaginalis subgroups in women with and without bacterial vaginosis
Source: BMC Infect Dis. 2017 Jun 5;17:394. doi: 10.1186/s12879-017-2501-y (PMC5460423; doi:10.1186/s12879-017-2501-y)
Supplement: Supplementary file 6 — Characterization of 109 vaginal samples by Nugent score, vly PCR for G. vaginalis, and clade-specific PCR. (PDF 142 kb) [file 12879_2017_2501_MOESM6_ESM.pdf]

**Additional file 6.** Characterization of 109 vaginal samples by Nugent score, *vly* PCR for *G. vaginalis*, and clade-specific PCR.

| **No.** | **Sample no.** | **Nugent score** | ***G. vaginalis* by *vly* PCR** | **Clade by PCR** |
|---|---|---|---|---|
| 1 | 001S1 | 9 | + | 1+2 |
| 2 | 002S1 | 8 | + | 1+2+4 |
| 3 | 003S1 | 1 | − | − |
| 4 | 004S1 | 1 | − | − |
| 5 | 005S1 | 3 | + | 2+4 |
| 6 | 006S1 | 4 | + | 1+4 |
| 7 | 007S1 | 8 | + | 1+2+4 |
| 8 | 008S1 | 6 | + | 4 |
| 9 | 009S1 | 3 | + | 4 |
| 10 | 011S1 | 9 | + | 1+4 |
| 11 | 012S1 | 0 | − | − |
| 12 | 013S1 | 1 | − | − |
| 13 | 014S1 | 9 | + | 2+4 |
| 14 | 015S1 | 9 | + | 1 |
| 15 | 017S1 | 4 | + | 1+2+4 |
| 16 | 019S1 | 8 | + | 4 |
| 17 | 020S1 | 4 | + | 2+4 |
| 18 | 021S1 | 3 | − | − |
| 19 | 022S1 | 0 | − | − |
| 20 | 023S1 | 2 | + | 1 |
| 21 | 005S2 | 2 | + | 4 |
| 22 | 024S1 | 4 | + | 1+2+4 |
| 23 | 025S1 | 3 | + | 4 |
| 24 | 026S1 | 8 | + | 1+2+3 |
| 25 | 027S1 | 4 | + | 1+3 |
| 26 | 028S1 | 2 | + | 3 |
| 27 | 029S1 | 5 | + | 2+3+4 |
| 28 | 030S1 | 1 | + | 3 |
| 29 | 031S1 | 3 | + | 4 |
| 30 | 032S1 | 1 | + | 1+4 |
| 31 | 033S1 | 1 | + | ND |
| 32 | 034S1 | 3 | + | 1 |
| 33 | 035S1 | 1 | + | ND |
| 34 | 036S1 | 0 | + | ND |
| 35 | 037S1 | 0 | + | 4 |
| 36 | 038S1 | 2 | + | 4 |
| 37 | 039S1 | 3 | + | 1+4 |
| 38 | 040S1 | 4 | + | 1+4 |
| 39 | 042S1 | 0 | + | 1+4 |
| 40 | 028S2 | 1 | + | 1+4 |
| 41 | 044S1 | 4 | + | ND |
| 42 | 045S1 | 0 | + | 4 |
| 43 | 046S1 | 7 | + | 1+2+4 |

| | | | | |
|---|---|---|---|---|
| 44 | 047S1 | 10 | + | 1+4 |
| 45 | 048S1 | 4 | + | 4 |
| 46 | 049S1 | 2 | + | 4 |
| 47 | 050S1 | 1 | + | ND |
| 48 | 051S1 | 4 | + | 1+2+4 |
| 49 | 052S1 | 4 | + | 1+4 |
| 50 | 053S1 | 10 | + | 4 |
| 51 | 054S1 | 1 | + | 4 |
| 52 | 056S1 | 6 | + | 1+4 |
| 53 | 057S1 | 10 | + | 1 |
| 54 | 058S1 | 9 | + | 1+2+4 |
| 55 | 059S1 | 10 | + | 1 |
| 56 | 060S1 | 10 | + | 2+4 |
| 57 | 063S1 | 8 | + | 1+2+4 |
| 58 | 064S1 | 7 | + | 1+2+4 |
| 59 | 065S1 | 9 | + | 1+2+4 |
| 60 | 066S1 | 2 | + | 1+3 |
| 61 | 067S1 | 1 | + | 1+2+4 |
| 62 | 068S1 | 4 | + | 4 |
| 62 | 069S1 | 3 | + | 4 |
| 64 | 070S1 | 2 | + | 1 |
| 65 | 071S1 | 4 | + | 2+4 |
| 66 | 072S1 | 0 | + | 1 |
| 67 | 073S1 | 0 | + | 4 |
| 68 | 074S1 | 0 | + | 1+4 |
| 69 | 076S1 | 6 | + | 1+2+4 |
| 70 | 077S1 | 4 | + | 4 |
| 71 | 078S1 | 5 | + | 2+4 |
| 72 | 079S1 | 4 | + | 1 |
| 73 | 080S1 | 1 | + | 1 |
| 74 | 081S1 | 2 | + | 1+4 |
| 75 | 082S1 | 9 | + | 1+2+4 |
| 76 | 030S2 | 0 | + | 1+2+4 |
| 77 | 058S2 | 5 | + | 4 |
| 78 | 084S1 | 9 | + | 1+2+4 |
| 79 | 085S1 | 2 | + | 1+4 |
| 80 | 086S1 | 6 | + | 1+2+3+4 |
| 81 | 087S1 | 7 | + | 1+2+4 |
| 82 | 088S1 | 10 | + | 1+2+3+4 |
| 83 | 089S1 | 8 | + | 1+4 |
| 84 | 090S1 | 5 | + | 1+2+4 |
| 85 | 091S1 | 1 | + | 2+4 |
| 86 | 092S1 | 3 | + | 1+2+3+4 |
| 87 | 093S1 | 3 | + | 2 |
| 88 | 094S1 | 2 | + | 1+2+3+4 |
| 89 | 095S1 | 3 | + | 1+2+3+4 |
| 90 | 096S1 | 7 | + | 1+2+3+4 |
| 91 | 097S1 | 0 | − | − |
| 92 | 098S1 | 8 | + | 1+2+3+4 |
| 93 | 099S1 | 3 | + | 1+2+3+4 |

| | | | | |
|---|---|---|---|---|
| 94 | 101S1 | 1 | + | 1+2+3+4 |
| 95 | 102S1 | 5 | + | 1+2+4 |
| 96 | 103S1 | 5 | + | 2+4 |
| 97 | 104S1 | 1 | + | 2+4 |
| 98 | 105S1 | 3 | + | 1+4 |
| 99 | 106S1 | 9 | + | 1+2+3+4 |
| 100 | 107S1 | 10 | + | 1+2+4 |
| 101 | 108S1 | 4 | + | 1+4 |
| 102 | 109S1 | 5 | + | 1+4 |
| 103 | 110S1 | 5 | + | 1+4 |
| 104 | 111S1 | 0 | + | 1+4 |
| 105 | 112S1 | 0 | + | 4 |
| 106 | 113S1 | 1 | + | 1+4 |
| 107 | 114S1 | 10 | + | 1+2+4 |
| 108 | 115S1 | 10 | + | 1+4 |
| 109 | 116S1 | 0 | + | 1+4 |
